# Fast EEG/MEG BEM-based forward problem solution for high-resolution head models

**DOI:** 10.1016/j.neuroimage.2024.120998

**Published:** 2025-01-01

**Authors:** William A. Wartman, Guillermo Nuñez Ponasso, Zhen Qi, Jens Haueisen, Burkhard Maess, Thomas R. Knösche, Konstantin Weise, Gregory M. Noetscher, Tommi Raij, Sergey N. Makaroff

**Affiliations:** a Dept. of Electrical and Computer Engineering, Worcester Polytechnic Institute, Worcester, MA, USA; b Technische Universität Ilmenau, Institute of Biomedical Engineering and Informatics, Ilmenau, Germany; c Methods and Development Group ‘Brain Networks’, Max Planck Institute for Human Cognitive and Brain Sciences, Leipzig, Germany; d Leipzig University of Applied Sciences (HTWK), Institute for Electrical Power Engineering, Leipzig, Germany; e Athinoula A. Martinos Center for Biomedical Imaging, Massachusetts General Hospital, Boston, MA, USA

**Keywords:** Electroencephalography (EEG), Magnetoencephalography (MEG), Forward problem, Inverse problem, Adaptive mesh refinement (AMR), Boundary Element Fast Multipole Method (BEM-FMM)

## Abstract

A fast BEM (boundary element method) based approach is developed to solve an EEG/MEG forward problem for a modern high-resolution head model. The method utilizes a charge-based BEM accelerated by the fast multipole method (BEM-FMM) with an adaptive mesh pre-refinement method (called *b*-refinement) close to the singular dipole source(s). No costly matrix-filling or direct solution steps typical for the standard BEM are required; the method generates on-skin voltages as well as MEG magnetic fields for high-resolution head models within 90 s after initial model assembly using a regular workstation. The forward method is validated by comparison against an analytical solution on a spherical shell model as well as comparison against a full *h*-refinement method on realistic 1M facet human head models, both of which yield agreement to within 5 % for the EEG skin potential and MEG magnetic fields. The method is further applied to an EEG source localization (inverse) problem for real human data, and a reasonable source dipole distribution is found.

## 1. Introduction

EEG source localization, or source reconstruction, is the process of finding locations, magnitudes, and orientations of neuronal sources within the cortex that reproduce electric potential or magnetic field quantities measured during experiment (Knösche and Haueisen, 2022). Multiple open-source software packages – including Brainstorm (Tadel et al., 2011), FieldTrip (Oostenveld et al., 2011), MNE (Gramfort et al., 2014), and EEGLab (Delorme and Makeig, 2004) – offer this capability via a boundary element method (BEM) using electric dipole source models. However, the computational models on which they operate are typically limited to three to four tissue layers derived from a subject’s MRI: scalp, outer skull, inner skull (or cerebrospinal fluid, CSF), and brain surface (CSF/gray matter interface). The tissue layers themselves are further restricted in resolution, usually comprising fewer than 10, 000 triangular surface elements each. These limitations are a direct consequence of the classic BEM formulation, which requires computing, storing, and solving a dense system matrix that grows with the square of the number of surface elements. For computational models that include additional layers or use denser resolution, the system matrix can quickly grow intractably large.

Recent progress in the charge-based BEM accelerated by the fast multipole method (FMM, with the corresponding matrix-free BEM abbreviated “BEM-FMM”) (Makarov et al., 2018) has made it possible to overcome the matrix storage limitation (Makarov et al., 2021) and thus consider head models with more layers – including cerebrospinal fluid (CSF), gray matter (GM) and white matter (WM) – and with facets numbering in the millions (Makarov et al., 2021; Wartman et al., 2024; Weise et al., 2022) or tens of millions (Makaroff et al., 2023). However, there exists another challenge specific to cortical dipole source models. For the inner (WM) and outer (GM) cortical surfaces, modern head segmentation pipelines produce meshes whose resolution is approximately the same as the distance between those surfaces and the source dipoles within the cortex. It has long been known (Ferguson and Stroink, 1997; Gençer and Tanzer, 1999) that the zeroth-order BEM cannot accurately model the response of singular sources at such short distances, no matter how many neighbor surface integrals are precomputed analytically. Therefore, adaptive mesh refinement (AMR) of the layers is necessary close to the sources (Wartman et al., 2024). While our previous AMR solution published in (Weise et al., 2022) and characterized in (Wartman et al., 2024) has demonstrated excellent numerical accuracy for this case, its long execution time (roughly 30 min) limits its practical applicability.

The present study introduces a much faster AMR method that is based on one-time mesh pre-refinement using the first, almost trivial, approximation to the solution of the BEM integral equation written in terms of the surface charge density. This method, which we name “*b*-refinement” for reasons explained in Section 2.3, reduces the forward solution time per dipole distribution by a factor of approximately 30 for a standard one-million-facet head model. A full solution including on-skin voltages as well as MEG magnetic fields can typically be computed within 90 s following the model’s initial assembly.

The study is organized as follows. Section 2 describes the idea of the method and its realization. It also describes three test cases: comparison with an analytical solution, comparison with the precise *h*-refinement-based AMR algorithm (Wartman et al., 2024; Weise et al., 2022) (which is considered as the ground truth) for realistic head models with three challenging dipole locations, and finally, application to practical source reconstruction for experimental EEG data on median nerve stimulation. Section 3 reports the obtained results in all three cases. The results are discussed in Section 4, and Section 5 concludes the study. The software is available to interested researchers via a GitHub repository (Wartman and Makaroff, 2024).

## 2. Materials and methods

### 2.1. Motivation for adaptive mesh refinement

**Fig. 1.**
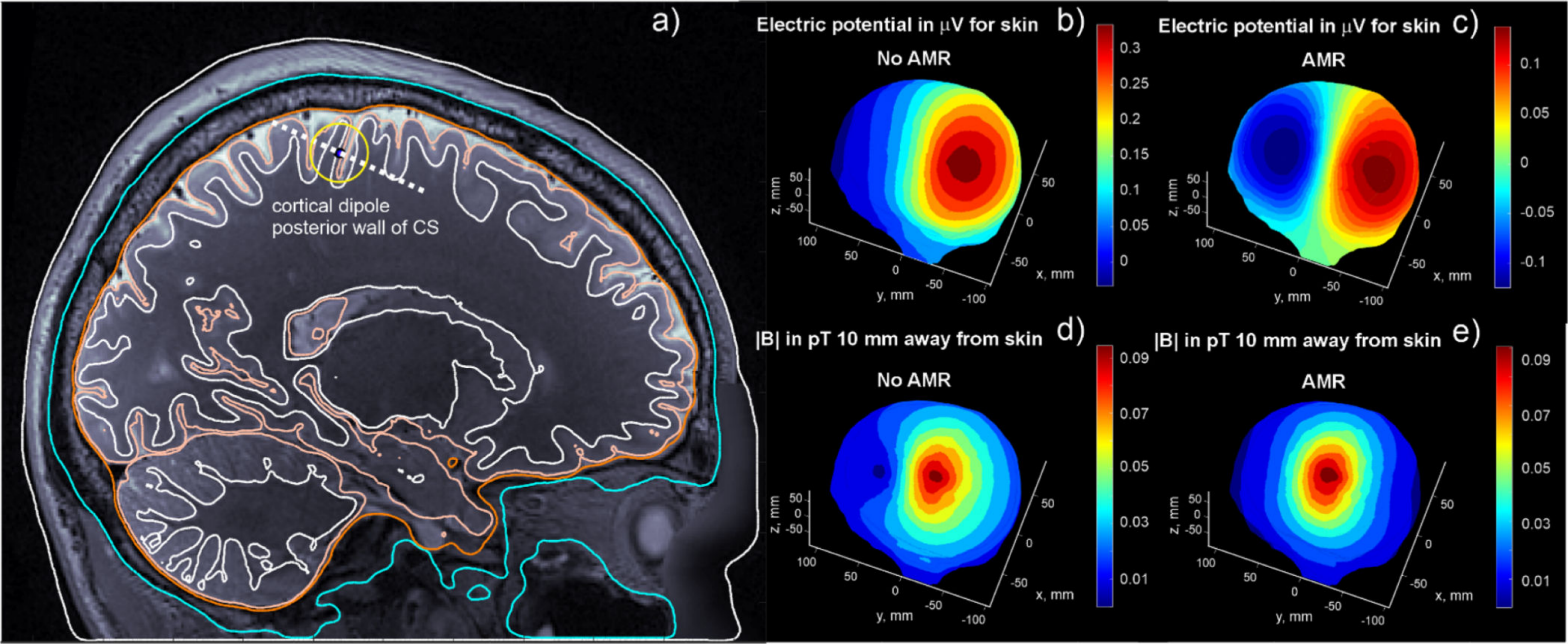
a) Cortical dipole position at the posterior wall of the central sulcus. The orientation is given by a dotted line. b,c) On-skin electric potential without (b) and with (c) adaptive mesh refinement. d, e) Magnitude of the magnetic field 10 mm away from the skin surface without (d) and with (e) adaptive mesh refinement. The cortical dipole has a moment of 4e-9 A·m. (Adapted from Fig. 6 of (Nuñez Ponasso et al., 2024), licensed under CC BY 4.0. The indicator of the dipole location has been modified from its original form.)

To motivate the need for adaptive mesh refinement in EEG/MEG forward modeling, we present a typical problem in Fig. 1a. A single cortical dipole is shown at the posterior wall of the central sulcus halfway between the gray matter (GM) and white matter (WM) surfaces for Human Connectome Project (HCP) Young Adult subject 110,411 (Van Essen et al., 2012). The dashed line indicates the dipole’s orientation. The head model was segmented by headreco (Saturnino et al., 2019) and comprises nested surface meshes for skin, skull, CSF, GM, WM, ventricles, and cerebellum. It has approximately 1 million triangular facets in total, and the average edge lengths for the GM and WM meshes are approximately 1.5 mm and 1.4 mm, respectively.

Such an edge length (triangle size) – the default computational resolution of the common segmentation packages FreeSurfer (FreeSurfer, 2012), headreco (Saturnino et al., 2019), and Charm (Puonti et al., 2020) – is typically greater than or equal to half of the cortical thickness (Cardinale et al., 2014; Seiger et al., 2018), and therefore also greater than the separation of the mid-surface dipole from either cortical boundary. As shown in Figs. 1b and 1d, this resolution is insufficient to enable a BEM-based solution to accurately model the singular dipole source very close to the nearest “large” triangles. Inaccurate predictions are obtained for both the on-skin electric potential (Fig. 1b) and off-skin magnetic field magnitudes (Fig. 1d) as compared to those for a sufficiently dense surface mesh in Figs. 1c and 1e, respectively. There, the refined facets in the vicinity of the source are approximately 10–20 times smaller than the separation distance between GM and WM shells (cortical thickness).

Therefore, regardless of the initial segmentation, mesh refinement must be performed for proper EEG/MEG source modeling (Wartman et al., 2024). Due to present computer hardware limitations, it is not practical to employ a uniform (global) mesh refinement scheme to achieve the required resolution. A local, and ideally adaptive (based on physically justified criteria) mesh refinement is therefore required for accurate EEG/MEG source modeling by BEM.

### 2.2. Our previous h-refinement method

The charge-based BEM equation for the surface charge density ρ(r) induced at all interfaces S of a piecewise-homogeneous multi-compartment head model is written in the following form (Makarov et al., 2018)

(1) $$\rho (r)=K(r)n(r)\cdot \int_{S}\frac{\rho \left(r^{\prime }\right)}{2\pi }\frac{r-r^{\prime }}{{\left|r-r^{\prime }\right|}^{3}}ds\left(r^{\prime }\right)+2\varepsilon_{0}K(r){\text{E}}^{i}(r)\cdot n(r),r\in S$$

where $K(r)=\frac{\sigma_{in}-\sigma_{out}}{\sigma_{in}+\sigma_{out}}$ is the electric conductivity contrast for the facet positioned at r, n(r) is the outer normal vector at the compartmental interfaces, and $\sigma_{in}$ and $\sigma_{out}$ are the conductivities of the materials just inside and outside (respectively) the interface at r. The induced charges are generated by the impressed or primary electric field ${\text{E}}^{i}(r)$ of a cortical current dipole (or a cluster thereof). A solution ρ(r) to Eq. (1) is found in an FMM-accelerated matrix-free algorithm via the Generalized Minimum Residual Method (GMRES), which takes its initial estimate for ρ(r) as:

(2) $$\rho (r)=2\varepsilon_{0}K(r){\text{E}}^{i}(r)\cdot n(r),r\in S$$

In our previous approach (Wartman et al., 2024; Weise et al., 2022), an *h*-refinement method was applied wherein facets of the model were selected for 4:1 barycentric subdivision according to the absolute value of total charge $q_{m}=\left|A_{m}\rho_{m}\right|$ upon them, where $A_{m}$ and $\rho_{m}$ denote the area and charge density, respectively, on the facet with index *m*. The solution was carried out in alternating steps of “solve” (by a full GMRES solution) and “refine” until a convergence criterion for relative inter-iteration change in skin potential was reached. The method achieved average errors smaller than 3 % in electric potential over the entire skin surface with respect to a globally refined reference solution (Wartman et al., 2024). However, the runtime – approximately 30 min per independent source in a standard 1M facet head model – became impractical when looping over multiple configurations of sources.

### 2.3. Concept of b-refinement

In this study, we suggest using the initial estimate for ρ(r) by Eq. (2) for an *a priori* mesh refinement, which is performed only once and before carrying out the iterative GMRES solution. While this initial estimate does not take into account the final charge redistribution due to self-interaction (i.e., the secondary electric field), it sufficiently identifies regions of the mesh where $q_{m}$ will have the maximum absolute values. These regions are indeed located close to the intracortical dipole source (s), but their exact topology depends on the compartmental conductivities and interfacial bends. This method is used strictly in place of *h*-refinement in this study.

We refer to the mesh refinement method based on Eq. (2) as “*b*-refinement” to highlight its association with the iterative solution of a well-conditioned system of linear equations in the form $x+\hat{A}x=b$ where the first approximation to x is x=b (the right-hand side). This is the form taken by Eq. (1) when discretized; for a detailed explanation, see (Wartman et al., 2024).

### 2.4. Mesh refinement strategy

First, Eq. (2) is used to find the approximate surface charge distribution on the conductivity interfaces. Following (Wartman et al., 2024; Weise et al., 2022), the facets having the largest *total* charges q are each subdivided into four congruent triangles whose edges are the halves of the original edges. To restore manifoldness (or “watertightness” of the mesh) after refinement, the border facets of the refinement region are also subdivided into two facets each.

An m-th facet is refined if its total charge $q_{m}$ satisfies the following inequality:

(3) $$\left|q_{m}\right|>k\times \bar{\left|q\right|}$$

where k is a constant on the order of 5–10 and the absolute mean charge value $\bar{\left|q\right|}$ is found over all facets. The refinement is performed iteratively according to the following steps: find a new initial charge distribution estimate following Eq. (2), select facets for refinement according to Eq. (3), subdivide those facets, and repeat. The number of necessary mesh refinement steps was empirically found to vary from 3 (the sphere model) to 4–5 (realistic head models). Surface-preserving Taubin smoothing (Taubin, 1995) with scale factors of −0.62 and +0.60 is additionally applied at every refinement step to reintroduce smooth curvature into the locally planar sub-regions created by the barycentric subdivision step. After application of *b*-refinement, the full charge solution is found by GMRES as usual, without further mesh refinement steps.

Appendix A presents a straightforward method for selecting *k* and the number of mesh refinement steps. For models of comparable size to those used here, these parameters can be selected within minutes for a specific modeling problem and family of models under study.

### 2.5. Forward problem validation example: dipole sources in a four-layer sphere

**Fig. 2.**
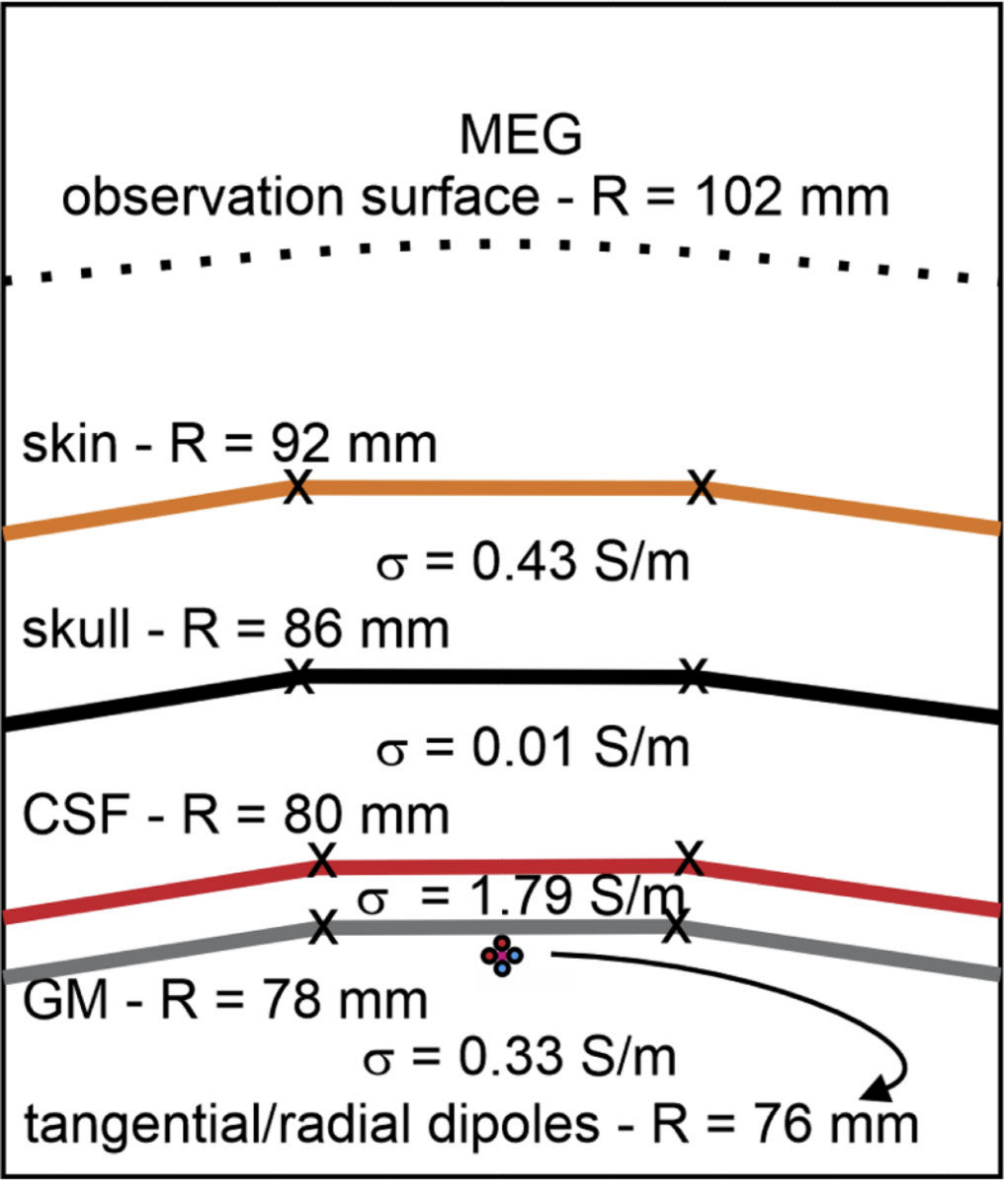
Model of the four-layer sphere used for comparison along with the dipole positions and conductivity values. From outside to inside, the layers of the model are as follows: MEG observation surface (dotted line), air/skin boundary (orange line), skin/skull boundary (black line), skull/CSF boundary (red line), and CSF/GM boundary (gray line). The tangential or radial dipole is located 2 mm inside the GM surface.

A classical EEG and MEG solution for a four-layer conducting sphere shown in Fig. 2 is analyzed first, similar to many other studies (cf. (Engwer et al., 2017; Piastra et al., 2018)). The four concentric spheres represent the air/skin boundary (“skin”), the skin/skull boundary (“skull”), the skull/CSF boundary (“CSF”), and the CSF/GM boundary (“brain”). To validate the *b*-refinement approach, we compare its output against known analytical solutions for EEG (Mosher et al., 1999; Zhang, 1995) and MEG (Sarvas, 1987) based on infinitesimal dipole sources within spherical shell models of this type. We test both tangential and radial dipoles located 2 mm inside the GM surface. To test different mesh resolutions, we create and clone nine sphere meshes with numbers of triangular facets ranging from approximately 6k to 400k using a high-quality surface mesh generator developed in (Persson and Strang, 2004; Persson, 2005).

### 2.6. Forward problem validation example: dipole sources in realistic head models

**Fig. 3.**
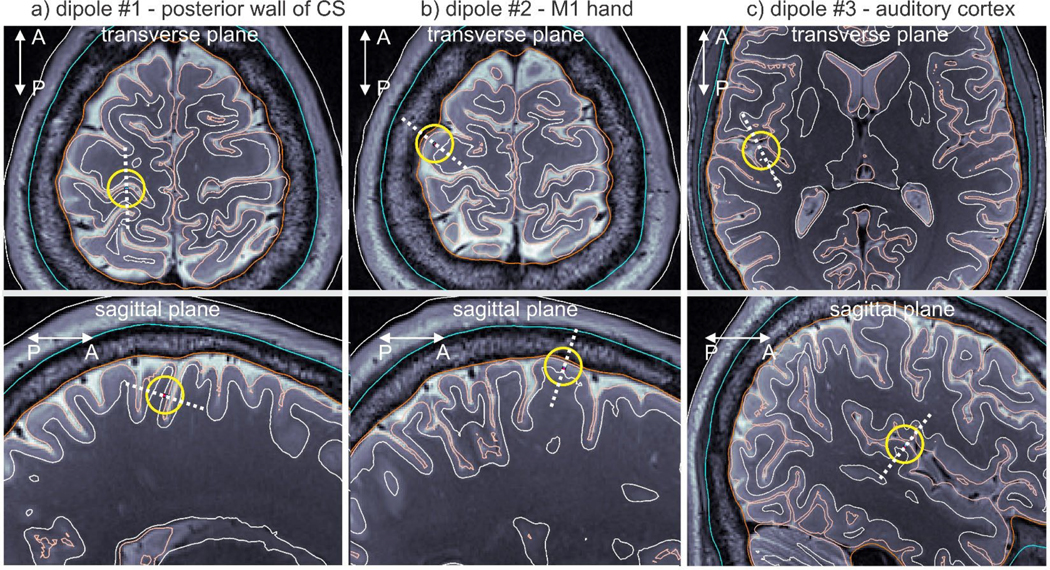
Three dipole positions used to test *b*-refinement in a realistic human head model (Connectome subject 110,411). Dipoles are located at the centers of the yellow circles; their orientations are given by dashed white lines. a) Position at the posterior wall of the central sulcus. b) Position within the $M_{1\text{HAND}}$ area. c) Position within the auditory cortex.

Headreco models for HCP Young Adult (Van Essen et al., 2012) subjects 110,411 and 120,111 are used with three dipole locations each: posterior wall of central sulcus, $M_{1\text{HAND}}$ area, and auditory cortex. The dipole locations for subject 110,411 are shown in Fig. 3. The material properties are those employed by the open-source software SimNIBS (Saturnino et al., 2019).

Both EEG (on-skin voltage) and MEG (vector magnetic field 10 mm away from skin) outcomes are compared across two solutions: the *b*-refinement method and the most accurate adaptive *h*-refinement solution (Weise et al., 2022), (Wartman et al., 2024), which shows excellent self-convergence but runs approximately 30 times slower. For further validation of the reference *h*-refinement solution for problems of this type, we refer interested readers to Section 2 and supplementary tables S13 and S14 of (Nuñez Ponasso et al., 2024). The standard L2-norm error and the RDM (relative difference measure) error (cf. (Wartman et al., 2024)) are applied over the entire skin surface/MEG observation points in both cases.

### 2.7. Inverse problem example: source localization with b-refinement using experimental data

A T1 MRI scan with 1-mm resolution of a healthy subject at A. A. Martinos Ctr. for Biomedical Imaging, Massachusetts General Hospital was followed by median nerve stimulation. Electrical stimuli over the median nerve at the right wrist were delivered to the subject using brief transcutaneous pulses every 1.5 s. The task was to respond to each stimulus by pushing a button with the left-hand index finger. This generates EEG evoked responses in $S_{1\text{HAND}}$ (primary somatosensory cortex contralateral to the nerve stimuli) and $M_{1\text{HAND}}$ at different latencies (Raij et al., 2008), (Liu et al., 2020).

**Fig. 4.**
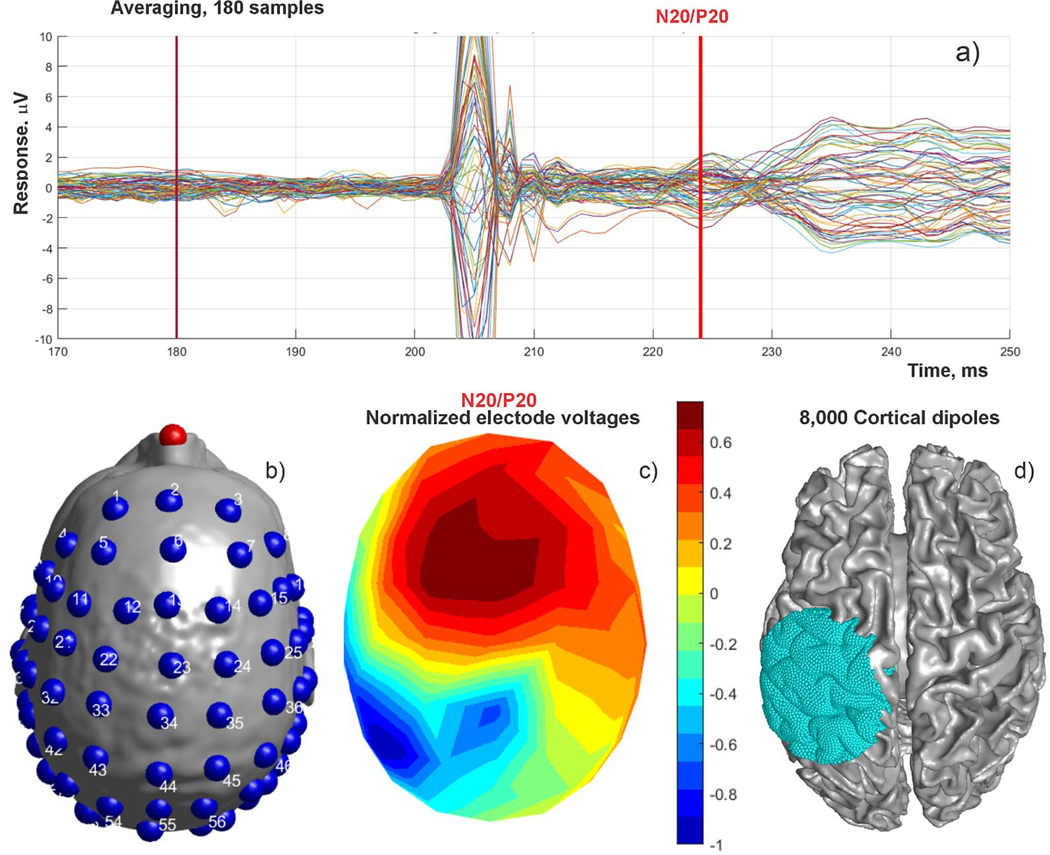
a) Electrode voltages and P20/N20 peak after median nerve stimulation of a heathy subject. b) Electrode positions (70 electrodes). c) Normalized on-skin voltage distribution for P20/N20 peak. d) Positions of 8000 cortical dipoles used for source localization.

The P20/N20 response peaking at about 20 ms after the stimulus in Fig. 4a was used for source localization, since at this latency its neuronal generators are well-known to be located in the posterior wall of the central sulcus in the Brodmann area 3b (Allison et al., 1991; Antonakakis et al., 2019). The recordings (3 runs of 80 trials each) were done at Massachusetts General Hospital using a 70-channel EEG system with electrode locations shown in Fig. 4b and the P20/N20 normalized electrode voltages shown in Fig. 4c. The head model and cortical surface reconstruction were obtained with SimNIBS headreco segmentation.

The *b*-refinement method was used to find forward solutions for 8000 cortical dipoles located in the vicinity of the expected neuronal response, at the mid-surface between gray and white matter. The dipole locations are schematically shown in Fig. 4d. The source localization problem was solved both by the standard Moore-Penrose pseudoinverse and by computing a minimum norm least-squares solution as a sanity check; both methods are implemented in base MATLAB. The electrode voltages are normalized by their self-variances. The goal of this experiment is to compare the known anatomical region of the neural activity with the modeling predictions.

### 2.8. Computing resources

The *b*-refinement method was developed, tested, and timed on a shared server running Windows Server 2022 Standard and MATLAB R2023a. The CPU is a 24-core Intel Xeon Gold 5317 operating at 3 GHz, and the system has 512 GB RAM installed. The software is parallelized and uses all available CPU cores. Approximately 14 GB RAM were required to run the computational method with *b*-refinement for the *headreco* models, measured in addition to the baseline memory used when MATLAB is open and idle with an empty workspace. All models were stored on and accessed from on-premises network storage.

## 3. Results

### 3.1. Forward problem validation example: dipole sources in a four-layer sphere

**Fig. 5.**
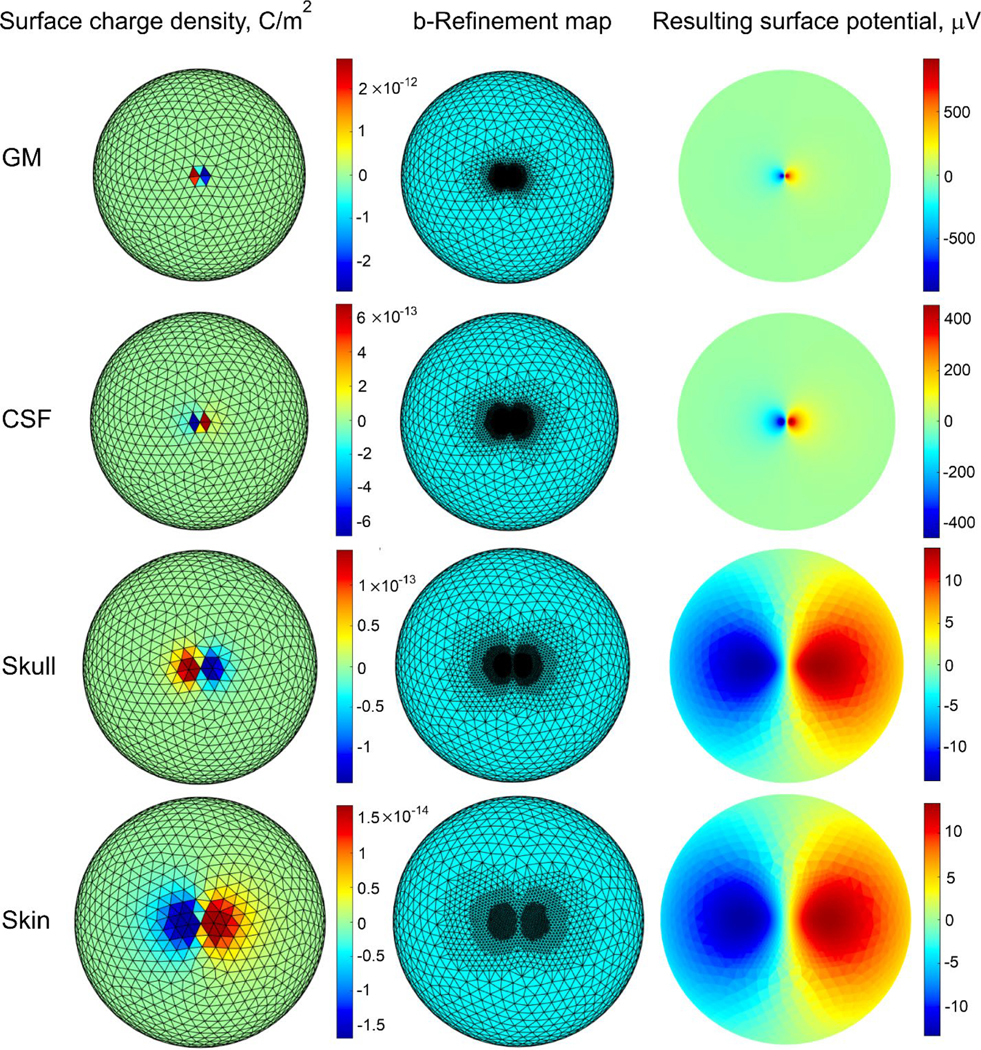
First column − initial surface charge distributions for the coarse four-layer sphere model from Fig. 2. Second column − the corresponding *b*-refined meshes obtained with four refinement steps for every shell. Note that the fourth step produces triangles that are barely visible on the figure. Third column − surface electric potential for every shell obtained after mesh refinement. All data are for the tangential electric dipole in Fig. 2 with the moment of 4e-11 A·m.

Fig. 5 demonstrates the solution process for a coarse four-layer sphere model. The source in this case is the tangential electric dipole in Fig. 2 with a moment of 4e-11 A/m. The figure’s first column shows, for each shell, the initial surface charge distribution estimates from Eq. (2) that were used as the starting point for AMR. The second column demonstrates the corresponding meshes obtained after four *b*-refinement steps, also for every shell. The figure’s third column demonstrates the resulting forward EEG solution (potential on every shell) after AMR is done.

**Fig. 6.**
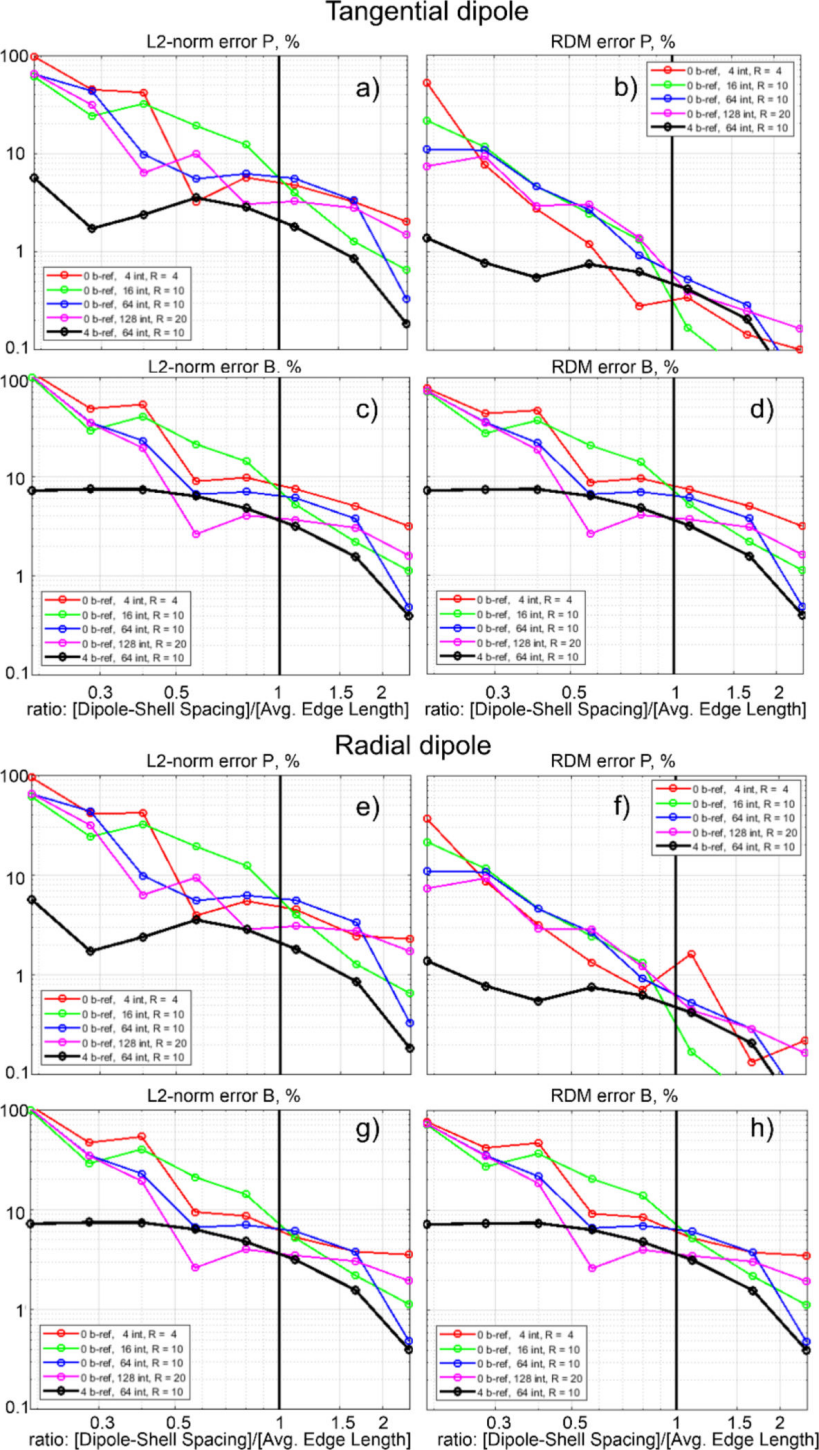
L2-norm and RDM error percentages between analytical and numerical solutions for the four-layer sphere model. The red, green, blue, and magenta curves are results without *b*-refinement and use analytical neighbor integration over 4, 16, 64, and 128 (respectively) neighbor triangles per triangle. The black curve shows results for b-refinement with four steps (levels) using 64 neighbor integrals.

The obtained solution was compared with the analytical solution as described in the previous section. Figs. 6a and b show the L2-norm/RDM error for electric potential over the entire outer shell (skin surface), and Figs. 6c and d show the 2-norm/RDM error in the vector magnetic field at a shell 10 mm from the skin surface. The results shown in Figs. 6 a–d are for the tangential electric dipole 2 mm inside the innermost shell (the GM surface), while Figs. 6 e–h present results for the radially-oriented electric dipole in the same location (cf. Fig. 2).

In every pane of Fig. 6, the argument is the dimensionless ratio of the dipole distance from the “brain” surface (2 mm) or the spacing between the “CSF” shell and the “brain” shell (also 2 mm) to the average edge length of the non-refined, original model mesh. The last number in every curve legend is a dimensionless radius of a sphere (in terms of average edge length) within which an analytical integration of the dipole’s primary electric field (Malmivuo and Plonsey, 1995) in Eq. (2);

(4) $$\varphi^{i}(r)=+\frac{1}{4\pi \sigma }m\cdot \nabla \left(\frac{1}{\left|r-r_{dipole}\right|}\right),{\text{E}}^{i}(r)=-\nabla \varphi^{i}(r)$$

over the planar surface triangles is performed instead of the center-point approximation used anywhere else. Here, $m=I_{0}d$ is the vector current dipole moment (A·m), d is the vector from the current sink $-I_{0}$ to the current source $+I_{0}$, and σ is the background conductivity of the medium where the dipole source is located.

In all cases, the solution with *b*-refinement achieves lower error than virtually all solutions without. The exception is the magenta curve representing a solution with 128 analytical neighbor integrals per triangle, which becomes impractical for large meshes due to the computational time required. As the ratio of dipole-shell spacing to mesh edge length decreases (i.e. the sphere meshes become coarser), only the *b*-refinement method reliably achieves errors smaller than 10 % in the quantities of interest. Supplementary convergence curves showing performance of *b*-refinement for varying numbers of neighbor integrals are presented in Appendix B.

### 3.2. Forward problem validation example: dipole sources in realistic head models

**Fig. 7.**
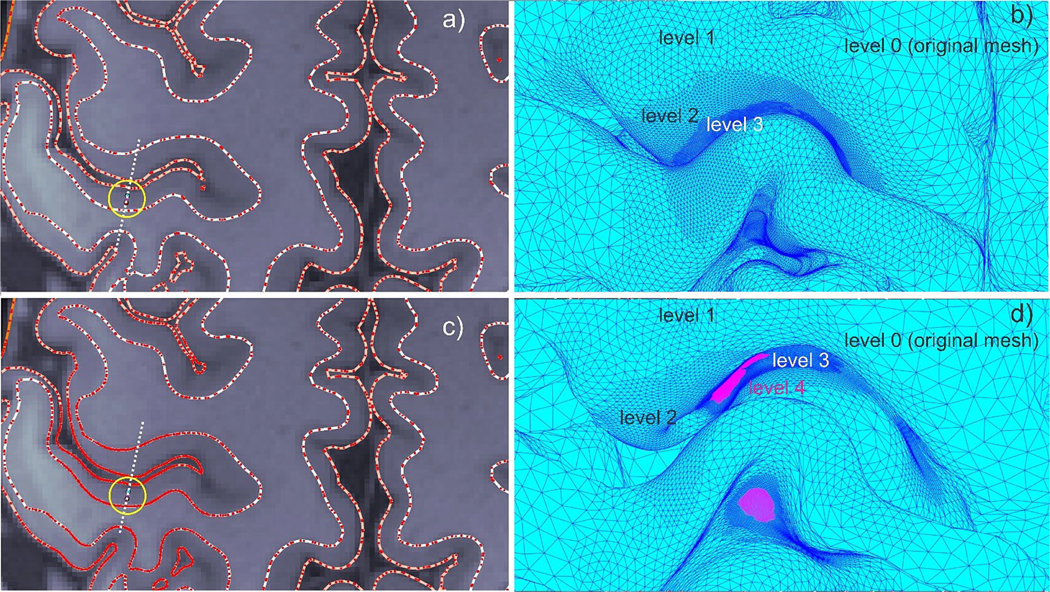
a) Original headreco segmentation superimposed onto T1 NIfTI data for Connectome subject 110,411. The dipole position at the posterior wall of the central sulcus is marked by a circle. Red dots indicate edge intersections with the transverse plane. b) *b*-refinement for the gray matter surface close to the dipole position after 4 refinement steps. Refinement level 4 is deeply inside the sulcus and is not visible. c) Same plot as in a), but after *b*-refinement with four steps. d) *b*-refinement for the white matter surface close to the dipole position after 4 refinement steps.

Fig. 7 demonstrates the *b*-refinement method outcome for the realistic head topology. Fig. 7a is the original headreco segmentation superimposed onto T1 NIfTI data for HCP subject 110,411. Fig. 7c shows the same segmentation after *b*-refinement; note the increased number of mesh edge intersections with the plane (marked by red dots) in the vicinity of the dipole source. Figs. 7b and d show the increased mesh density at the GM and WM surfaces, respectively. The skin surface is not refined for the present dipole position when four refinement steps are used. The *b*-refinement leads to a moderate total mesh size increase of approximately 10 % (1.04 M to 1.14 M facets) in the present case.

**Fig. 8.**
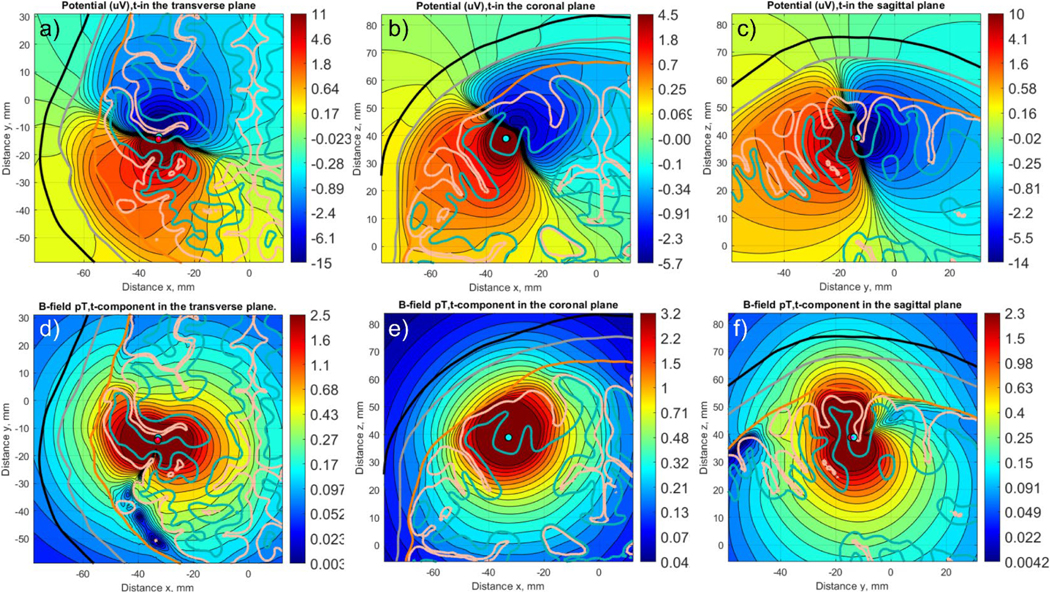
a-c) electric potential distribution in three principal planes for Connectome subject 120,111. The cortical dipole is located at the posterior wall of the central sulcus. d-f) magnetic field (flux) magnitude distribution for the same cortical dipole in three principal planes. Note that a logarithmic scale is used in both cases.

Table 1 reports averaged L2-norm and RDM relative differences for the two head models and three dipole positions (previous section, Fig. 3) between the *b*-refinement method with four levels of refinement and the reference AMR solution (Nuñez Ponasso et al., 2024; Wartman et al., 2024) based on *h*-refinement. The maximum difference was observed for the dipole in the auditory cortex (dipole 3) of subject 110,411. For the EEG electric potential, the differences are computed for the entire skin surface. For the MEG vector magnetic field (magnetic flux) **B**, the differences are computed 10 mm away from the skin surface in the radial direction. Fig. 8 demonstrates the dipole fields within the cortical and extracerebral compartments for subject 120,111.

Table 2 reports timing data for each phase of the BEM-FMM augmented by *b*-refinement, averaged over 3 runs each, for HCP subjects 110,411 and 120,111. Simulations for these models took 81 and 77 s end-to-end, respectively, on average.

### 3.3. Inverse problem example: source localization from b-refinement using experimental data

The output of the source localization problem described in Section 2.7 is a set of 8000 weights (or “strengths”), one per independent neuronal source dipole model, indicating the activity of each possible neuronal source at the selected instant in time (the P20/N20 peak). Sources with small weights are inactive; sources with large (absolute) weights are active. Sources with negative weights have actual current flows that are directed oppositely to their assumed orientations. The dipoles in this study are directed from the WM surface to the GM surface; negative weights therefore indicate dipoles that are found to point from GM to WM instead.

**Fig. 9.**
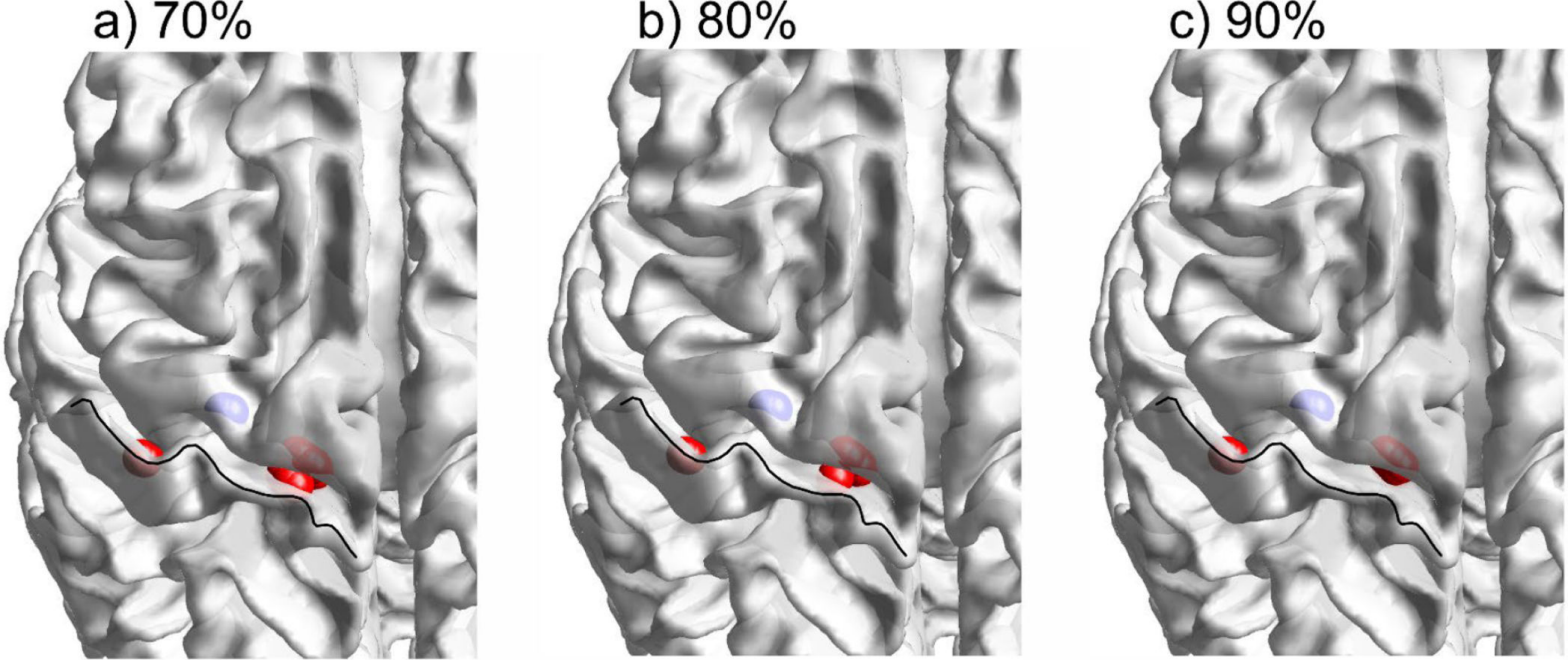
Reconstructed dipole strength density for the experimental data on median nerve stimulation at 20 ms post-stimulus. Red spheres indicate cortical dipoles with the maximum positive strength (directed from white matter to gray matter) while blue dots indicate cortical dipoles with the maximum negative strength (directed oppositely). a-c): threshold values of dipole strength are set to the 70th, 80th, and 90th percentiles, respectively. The crown of the postcentral gyrus is indicated by a black curve in every pane. The apparent invariance of the distribution with respect to the choice of threshold indicates strong activity in three quite focal regions at this time point.

Fig. 9 displays the reconstructed dipole strength density found for the median nerve stimulation experimental data recorded at the P20/N20 peak. In each pane, dipoles whose strength exceeds a given threshold – 70th, 80th, or 90th percentiles – are marked by red spheres (positive magnitudes) or blue spheres (negative magnitudes). The crown of the postcentral gyrus is indicated by a black curve in every pane. The apparent invariance of the distribution with respect to the choice of threshold indicates strong activity in three quite focal regions at that time point.

## 4. Discussion

### 4.1. Forward problem validation example: dipole sources in a four-layer sphere

The results in Fig. 6 demonstrate that the *b*-refinement method leads to much lower and very consistent computational errors for relatively coarse meshes, when the ratio of the dipole distance from the nearest shell to the average edge length of the non-refined (original) model mesh is less than one (Knösche and Haueisen, 2022). This condition is present in the realistic head models used in the rest of this study. A larger number of neighbor integrals computed analytically also reduces the error, but this approach becomes impractical due to the large storage and extensive precomputations necessary.

### 4.2. Forward problem validation example: dipole sources in realistic head models

Table 1 indicates that the RDM error for both EEG and MEG forward problems does not exceed 2 % on average when *b*-refinement is applied with a relatively small number of steps. Similarly, the average 2-norm error does not exceed 4.5 %. This is certainly acceptable in practical applications. However, the large peak deviation in skin potential in Table 1 indicates that, in some cases, the secondary interactions do have a critical impact on the required mesh discretization beyond that predicted by the incident field. Section 4.4 will propose a possible means of increasing the accuracy further for these cases.

Notably, the *b*-refinement results in a very modest overall mesh size increase of 10–15 % for a point dipole or a small cluster of closely spaced dipoles up to 5–7 mm in diameter. Furthermore, Fig. 8 demonstrates that the dipole field can be substantially distorted by nearby white and gray matter interfaces. This underscores the importance of using high-resolution models and adaptive mesh refinement for source reconstruction.

### 4.3. Inverse problem example: source localization from b-refinement using experimental EEG data

The source reconstruction maps in Fig. 9 indicate that the present approach provides localization results for median nerve stimulation that agree with the experimental predictions for neuronal generators (Allison et al., 1991), (Antonakakis et al., 2019). All three panes in Fig. 9 predict source locations in the posterior wall of the central sulcus or at its bottom, in the Brodmann area 3b. Those are the red spheres in Fig. 9.

At the same time, dipolar sources of opposite polarity can also be predicted at the anterior wall of the central sulcus as shown by a blue cluster in Fig. 9. This result is to be expected since the anterior and posterior walls are very close to each other, implying that a dipole in the anterior wall with an opposite orientation would generate nearly the same EEG response as its counterparts in the posterior wall. A (small) change in the source location has apparently little effect on the ill-posed EEG inverse problem. In any case, the solution remains stable and meaningful with respect to the source strength threshold − all three panes in Fig. 9 are quite similar to each other.

### 4.4. Extensions of the method and future work

In this work, the *b*-refinement method has been used as a standalone replacement for *h*-refinement. An approach that combines the two methods would be conceptually straightforward to implement, involving an initial pass by the *b*-refinement method followed by perhaps 1–2 passes of *h*-refinement. Such an approach may achieve a balance between the efficient execution time of the *b*-refinement and the excellent accuracy of the *h*-refinement. Further, while the current implementation of the method is readily applicable to problems with sources separated from the model (e.g. EEG and TMS (Makarov et al., 2018)), its usefulness is limited in problems such as TES (Weise et al., 2022), where the right-hand-side of Eq. (1) is zero everywhere except for a small number of electrode facets. In this case, the initial charge estimate *b* would need to be treated as a distinct set of sources, and the initial refinement step would need to be performed based on some *b’* calculated from those initial charges.

The *b*-refinement approach is also applicable to head (or other) models with topologies not presented in this work, including highresolution skull models that distinguish compact bone from spongy bone and/or models that include detailed meningeal layers (Stenroos et al., 2014; Wartman et al., 2024; Weise et al., 2022).

## 5. Conclusion

The *b*-refinement method for forward EEG and MEG problems introduced in this study has been verified both theoretically and experimentally. This method, in conjunction with the matrix-free boundary element fast multipole method (BEM-FMM), allows us to solve a forward problem for a single dipole or a compact dipole cluster within 90 s when a modern detailed (ca. 1M facet) head model is used. All major computational steps – model assembly from input surface meshes, nearfield interaction integral evaluation, mesh refinement, interfacial charge solution, and calculation of on-skin voltages as well as MEG magnetic fields – are included in the 90-second estimate.

## Supplementary Material

1

2

## Figures and Tables

**Table 1:** L2-norm and RDM relative differences for two head models and three dipole positions (Fig. 3) between the *b*-refinement method with four levels of refinement and the reference AMR solution (Wartman et al., 2024), (Nuñez Ponasso et al., 2024). For the EEG electric potential, the differences are computed for the entire skin surface. For the MEG vector magnetic field (magnetic flux) **B**, the differences are computed at a surface 10 mm away from the skin surface (in the radial direction). The peak relative difference occurred for the dipole in the auditory cortex (dipole 3) of subject 110,411.

Quantity	Mean relative difference (2 subjects, 3 dipoles)	Peak relative difference (110,411, auditory cortex)

Potential error - RDM	1.86 %	4.03 %
Potential error - L2-norm	4.27 %	8.64 %
**B** Field error - RDM	1.79 %	2.47 %
**B** Field error - L2-norm	3.68 %	4.99 %

**Table 2:** Time for each phase of BEM-FMM with *b*-refinement for Connectome subjects 110,411 and 120,111 in addition to the 4-layer sphere model, averaged over three trials each.

Phase	Time (s): 110,411 (1.04 M facets initial, 1.14 M facets refined)	Time (s): 120,111 (0.83 M facets initial, 0.93 M facets refined)	Time (s): Sphere model (11 k facets initial, 26 k facets refined)

STL file import and initial model assembly	4.4	2.7	0.17
*E*^*i*^ calculation	1.2	1.1	0.04
*b*-refinement	6.5	5.6	1.38
Nearfield interaction integral computation	19.8	15.5	5.09
Iterative solution for *c*	43.7	46.2	12.10
Field calculation (*E* and *B*) at observation points	5.6	6.0	0.58
**Total**	**81.2**	**77.1**	**19.36**

## Data Availability

The software described is available for download via a GitHub repository: https://github.com/wiwartman/BEM-FMM-with-B-Refinement-for-EEG/. The boundary element method accelerated by the fast multipole method (BEM-FMM) and augmented by the proposed *b*-refinement method is available for download under a GNU GPL v3.0 license at https://github.com/wiwartman/BEM-FMM-with-B-Refinement-for-EEG/. Three examples are included corresponding to the validation examples in Sections 2.5, 2.6, 3.1, and 3.2.
