# Supplementary material for: Fast EEG/MEG BEM-based forward problem solution for high-resolution head models
Source: Neuroimage. Author manuscript; Available in PMC 2025 Mar 26. (PMC11941539; doi:10.1016/j.neuroimage.2024.120998)
Supplement: 1 [file NIHMS2051060-supplement-1.docx]

**Appendix A**

Section 2.4 mentions an empirical tuning method for the mesh refinement selection parameter *k* as well as the number of mesh refinement steps *n*. This appendix provides a simple example of such a tuning method. It is also extensible to other parameters in the toolkit, such as the number of neighbor integrals, FMM precision, GMRES convergence tolerance, etc.

This exercise uses the example in the folder “Example03_Connectome122620” available at [32] of the main text. The model in use is a 1 M facet human head model with a single cortical dipole. The script Model\addAMR has been modified to check for external parameter declarations of the AMRSTEPS and factor variables. The high-level tuning script wrapper_testAMRParameters is reproduced in full below.

clear all; %#ok

% First run: solution with first selection of AMR

ext_AMRSTEPS = 1; %#ok

ext_k = 10; %#ok

wrapper_execute;

soln_amr1.SurfaceP = PtotSkin;

soln_amr1.SurfaceB = Btotal;

% Second run: solution with second selection of AMR

ext_AMRSTEPS = 6;

ext_k = 6;

wrapper_execute;

soln_amr2.SurfaceP = PtotSkin;

soln_amr2.SurfaceB = Btotal;

% Calculate and display 2-norm error between solutions

diffP = norm(soln_amr1.SurfaceP - soln_amr2.SurfaceP)/norm(soln_amr2.SurfaceP);

diffB = norm(soln_amr1.SurfaceB - soln_amr2.SurfaceB)/norm(soln_amr2.SurfaceB);

disp(['DiffP: ' num2str(diffP*100) '%']);

disp(['DiffB: ' num2str(diffB*100) '%']);

The script runs the BEM-FMM with *b*-refinement twice, once for each user-requested combination of parameters. It calculates simple 2-norm relative differences between the skin potentials (diffP) and the MEG magnetic fields (diffB) for the two runs. The script executes within 3-5 minutes on the hardware reported in Sec. 2.8. Table A1 below reports results for several combinations of parameters tested in the first run, while the parameters in the second run are held constant at $k=6$ and AMRSTEPS = 6.

For models where “reference case” choices of *k* and AMRSTEPS are not known *a priori*, an iterative process can be used. In this case, the second run should always use incrementally more precise values of *k* and AMRSTEPS than the first, and precision in all runs should be increased uniformly until the relative changes in potential and magnetic field drop below a user-accepted threshold (i.e., the solution converges).

Table A1: 2-norm error in potential and magnetic field under different selections of *k* and AMRSTEPS. The reference solution uses *k* = 6 and AMRSTEPS = 6.

| Tested *k* | Tested AMRSTEPS | P 2-norm relative difference | B 2-norm relative difference |
| --- | --- | --- | --- |
| 10 | 1 | 49.98% | 31.26% |
| 10 | 2 | 2.75% | 2.37% |
| 10 | 4 | 0.76% | 1.22% |
| 8 | 1 | 49.86% | 31.19% |
| 8 | 2 | 2.58% | 2.22% |
| 8 | 4 | 0.77% | 1.22% |
| 6 | 1 | 50.08% | 31.37% |
| 6 | 2 | 2.81% | 2.33% |
| 6 | 4 | 0.80% | 1.19% |
