# Supplementary material for: Fast EEG/MEG BEM-based forward problem solution for high-resolution head models
Source: Neuroimage. Author manuscript; Available in PMC 2025 Mar 26. (PMC11941539; doi:10.1016/j.neuroimage.2024.120998)
Supplement: 2 [file NIHMS2051060-supplement-2.docx]

**Appendix B**

The error curves given in Fig. 6 for the four-layer sphere model of Sec. 2.5 include only one curve where *b*-refinement was used. This appendix provides a brief survey of performance of *b*-refinement for the 4-layer sphere under different numbers of refinement steps and neighbor integrals.

In Fig. B1, the number of neighbor integrals per facet is held constant in each sub-plot while the number of *b*-refinement steps is varied from 0 to 6. L2 errors are reported for the skin surface potential (P) and the MEG surface magnetic field (B). The numbers of neighbor integrals tested are 4, 16, 64, and 128. As in the main text, the x-axis is the dimensionless ratio of dipole-shell spacing to average mesh edge length, and the y-axis is error reported as a percentage.

In Fig. B2, the number of *b*-refinement steps is held constant in each sub-plot while the number of neighbor integrals is varied from 4 to 128. The numbers of *b*-refinement steps tested are 0, 4, and 6. The curves in Fig. B2 are the same as those in Fig. B1 and retain their respective line styles (corresponding to number of *b*-refinement steps) and colors (corresponding to number of neighbor integrals per facet).

Figs. B1 and B2 demonstrate that an appropriate selection of number of neighbor integrals can enable order-of-magnitude improvements from *b*-refinement when the initial mesh is coarse (dipole spacing ratio > 1). The accuracy improvement due to *b*-refinement is best characterized by Fig. B1e-h, while the dependence on an appropriate choice of neighbor integrals is clearly illustrated in Fig. B2c-f. Fig. B2a-b demonstrate that an increased number of neighbor integrals alone cannot compensate for a coarse mesh.


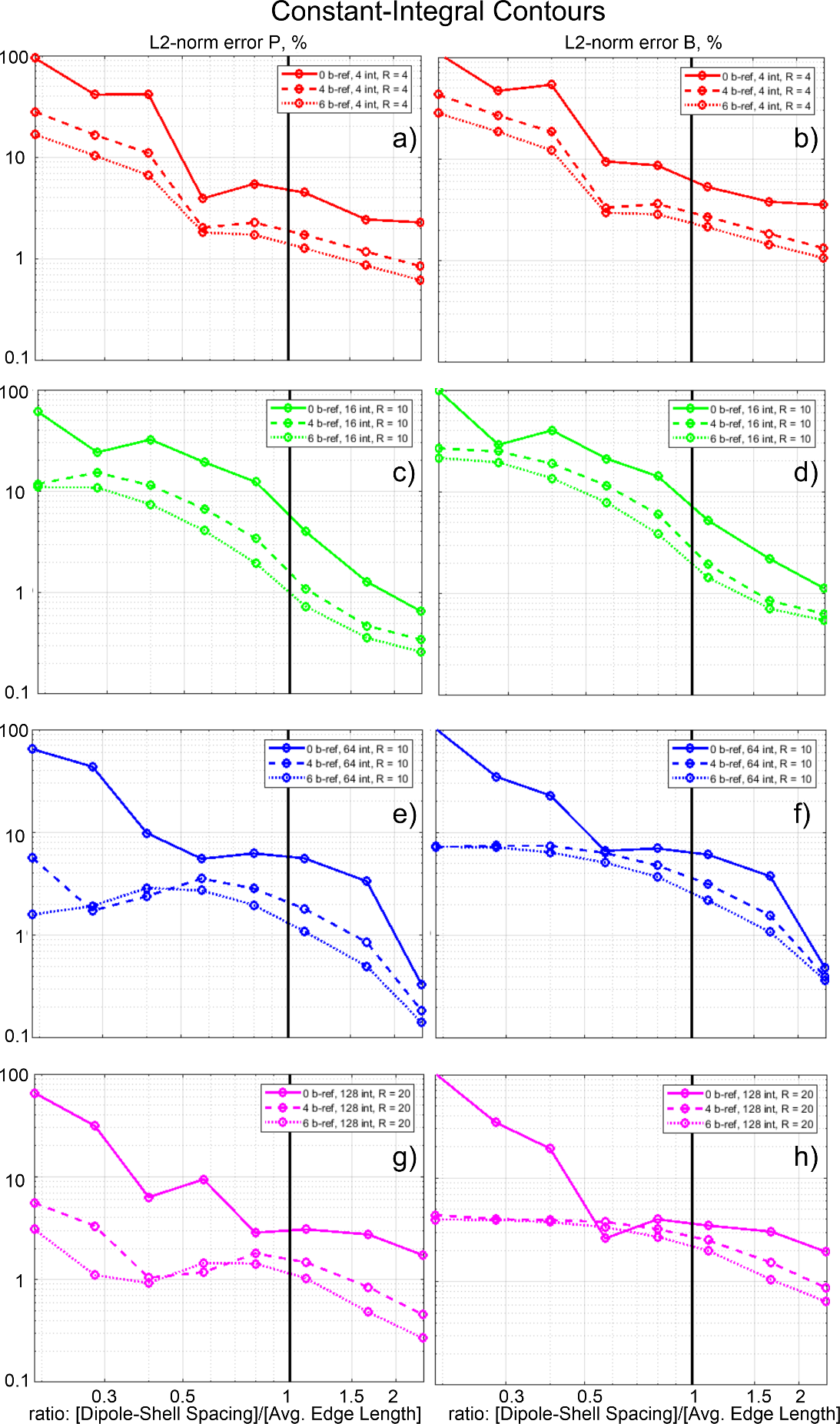


Fig. B1: Constant-integral error convergence curves for the four-layer sphere model of Sec. 2.5. The argument, shared among all plots, is the dimensionless ratio of dipole-shell spacing to average mesh edge length. The left column reports L2 error in skin surface electric potential while the right column reports L2 error in MEG surface magnetic field. B1a-b: 4 neighbor integrals. c-d: 16 neighbor integrals. e-f: 64. g-h: 128.


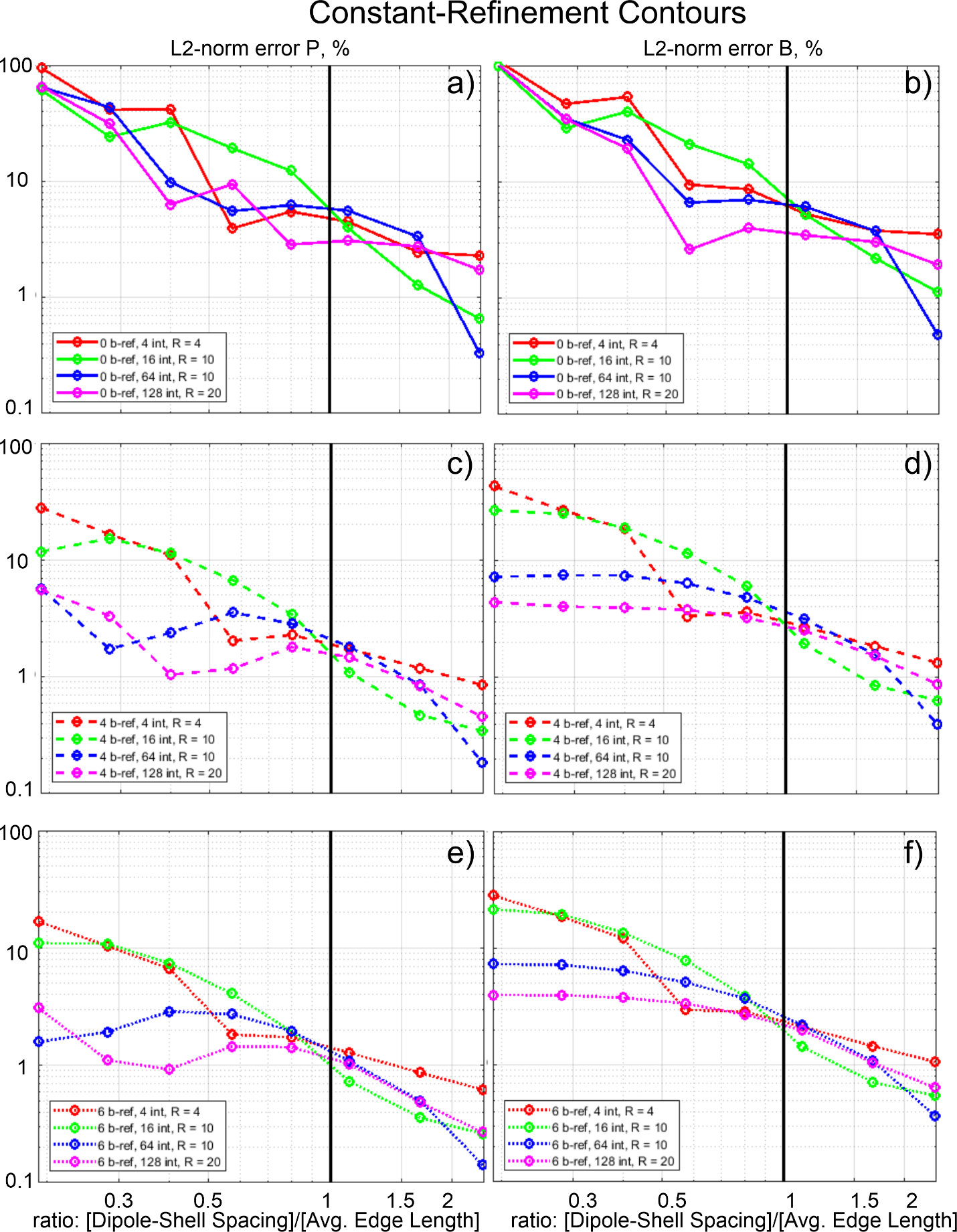


Fig. B2: Constant-refinement convergence curves for the four-layer sphere model of Sec. 2.5. The argument, shared among all plots, is the dimensionless ratio of dipole-shell spacing to average mesh edge length. The left column reports L2 error in skin surface electric potential while the right column reports L2 error in MEG surface magnetic field. B2a-b: 0 *b*-refinement steps. c-d: 4 *b*-refinement steps. e-f: 6 steps.
